# The Current Status of Research on the Integration of Sexual and Reproductive Health and HIV Services

**DOI:** 10.1111/sifp.12024

**Published:** 2017-05-10

**Authors:** Charlotte E. Warren, Susannah H. Mayhew, Jonathan Hopkins

## Abstract

Integration of services for sexual and reproductive health (SRH) and HIV has been widely promoted globally in the belief that both clients and health providers benefit through improvements in quality, efficient use of resources, and lower costs, helping to maximize limited health resources and provide comprehensive client‐centered care. This article builds on the growing body of research on integrated sexual SRH and HIV services. It brings together critical reviews on issues within the wider SRH and rights agenda and synthesizes recent research on integrated services, drawing on the Integra Initiative and other major research. Unintended pregnancy and HIV are intrinsically interrelated SRH issues, however broadening the constellation of services, scaling up, and mainstreaming integration continue to be challenging. Overcoming stigma, reducing gender‐based violence, and meeting key populations’ SRH needs are critical. Health systems research using SRH as the entry point for integrated services and interaction with communities and clients is needed to realize universal health coverage.

In countries where the risks of both unintended pregnancy and HIV transmission are high, integration of sexual and reproductive health (SRH) and HIV services makes sense. Adequate integrated SRH services for all men and women includes treatment to extend the lives and improve the well‐being of people living with HIV and to prevent further transmission of the virus, as well as meeting wider family planning (FP) and maternal and SRH needs. Men, women, and adolescents attending non‐HIV services would be offered HIV counseling, testing, and treatment. SRH‐HIV integration aims to coordinate comprehensive healthcare responses to meet multiple and related client needs, and exploit intersections in service delivery to achieve wide‐reaching reproductive health goals in a cost‐efficient way (Church and Mayhew 2009; Spaulding et al. 2009; Wilcher et al. 2013a; Obure et al. 2015). This Introduction surveys the latest findings on SRH‐HIV integration, drawing heavily from the Integra Initiative as well as the findings of the five other articles in this special issue, ending with a consideration of the research needs and gaps that remain.

An integrated SRH‐HIV health system manages and delivers health services so that clients receive a continuum of preventative and curative services according to their needs over time and across different levels of the health system (WHO 2008). Since the International Conference on Population and Development in 1994, the integration of HIV services into SRH services has been increasingly promoted (WHO, UNFPA, IPPF, and UNAIDS 2005; WHO, UNFPA, IPPF, UNAIDS, and UCSF 2009; WHO, USAID, and FHI 2009). The provision of integrated services has the potential for both clients and providers to benefit through improvements in quality; uptake and efficient use of resources; lower costs; and reduced duplication of efforts, helping maximize the impact of limited health resources and provide a more comprehensive package of health care for its users (Sweeney et al. 2012; Lassi et al. 2013; Baumgartner et al. 2014; Obure et al. 2015; Mak et al. 2016). Integration can yield positive effects on service quality as well as reducing unmet need for contraceptive use, antiretroviral therapy (ART) in pregnancy for women living with HIV, and HIV testing (Warren, Abuya, and Askew 2013; Integra Initiative 2015c; Mutemwa et al., forthcoming).

Much has been achieved since the Glion Call to Action on Family Planning and HIV/AIDS in Women and Children (WHO and UNFPA 2006) and the New York Call to Commitment: Linking HIV/AIDS and SRH and Rights (UNFPA 2004). Only in the last decade, however, has a sufficient body of research and integration policies supported integrating or linking SRH and HIV services nationwide in low‐ and middle‐income countries experiencing generalized HIV epidemics, with research primarily in sub‐Saharan Africa (for example, Gillespie et al. 2009; Warren et al. 2012; Grossman et al. 2013; Johnstone et al. 2013; Hope et al. 2014). Literature reviews have focused on the benefits of HIV and FP linkages, specifically increasing contraceptive uptake among clients with HIV who do not wish to become pregnant (Church and Mayhew 2009; Spaulding et al. 2009; Wilcher, Cates, and Gregson 2009; Brickley et al. 2011; Wilcher et al. 2013a; Wilcher et al. 2013b; Kimani et al. 2015a). There has been limited evaluation in the research literature, however, on models of integrating HIV with broader SRH services, or on issues and gaps in our understanding of the processes needed at the facility level to achieve successful delivery of integrated care. Consequently, despite progress in mainstreaming SRH‐HIV in a number of countries’ policies and strategies, integrating such obviously linked programs has proven difficult, especially at the point of service delivery.

Sustaining promising interventions is challenging, even just ensuring that women are offered more than one service every time they visit, regardless of entry point (Birdthistle et al. 2014). Despite unintended pregnancy and HIV being intrinsically interrelated SRH issues, many health facilities do not provide health services that simultaneously address both in meaningfully integrated ways. As a result, women often must seek services from separate providers, waiting on different lines in different parts of a facility, or travel to entirely separate facilities; health facilities often experience staff shortages and inadequate supplies (Hargreaves et al. 2016). Few differences have been detected in reproductive behaviors as a function of HIV sero‐status, but substantial proportions (50–90 percent) of women living with HIV continue to have unmet need for family planning and other reproductive health services (Homsy et al. 2009; Smee et al. 2011; Lindegren et al. 2012; Schwartz et al. 2012; Wilcher et al. 2013a). Qualitative studies show that women living with HIV receive limited and fragmented FP care within their HIV services (Church et al. 2014; Colombini et al. 2016a). Providers’ attitudes may discourage further fertility and stress condom‐based contraception, resulting in unintended pregnancies (Myer et al. 2010; Warren, Abuya, and Askew 2013; Church et al. 2014), which poses serious challenges to successful formulation and implementation of reproductive goals among sero‐positive women (Stuart 2009; IATT 2011; Lindegren et al. 2012; Newmann et al. 2013). Where SRH consultations have served as entry points for HIV prevention, treatment, and care in medium‐ and high‐HIV prevalence contexts, little was known about existing levels of integration, particularly in public‐sector facilities, or how provision could be improved and scaled up.

## RECENT RESEARCH INITIATIVES ON SERVICE INTEGRATION

Three major initiatives have added considerably to the research and practice literature over the past decade. First, between 2008 and 2013, the Integra Initiative, a multi‐centered nonrandomized trial, explored a range of issues relating to the integration of health services. The largest complex evaluation of its kind, Integra sought to determine the impact of different models of SRH‐HIV service integration on service and health outcomes in Kenya and Swaziland (Warren et al. 2012; Mayhew et al. 2016). Through Integra, three organizations—the International Planned Parenthood Federation, the Population Council, and the London School of Hygiene & Tropical Medicine—worked together to generate evidence on the feasibility, effectiveness, cost, and impact of different models for delivering integrated SRH‐HIV care through mainstream SRH services in areas with high and medium HIV prevalence in sub‐Saharan Africa. Integration was defined as services for HIV/sexually transmitted infections (HIV/STI) (including counseling, testing, and treatment) *and* reproductive health (RH) services (including FP, antenatal, and postnatal) for a client at the same facility, on the same day. Multiple benefits have been claimed for this type of integration, yet the evidence has been, at best, inconsistent (Church and Mayhew 2009; Kennedy et al. 2010; Sweeney et al. 2012; Warren et al. 2012; Mayhew et al. 2016). Integra also investigated the effect of integration on stigma, unintended pregnancy, and HIV risk behavior (Warren et al. 2012).

The initial evaluation design for the Integra Initiative comprised intervention and matched comparison facilities. However, maintaining the comparison sites was difficult in an ever‐changing policy environment, with other development partners supporting SRH and HIV services at county or regional levels. Both Kenya and Swaziland formally adopted integration policies in their national HIV strategies in late 2009, for comprehensive HIV prevention (counseling and testing) and treatment for clients seeking maternal and child health (MCH) and FP services (Obure et al. 2015). This “real world” setting in which Integra was implemented presented challenges for measuring the effect of the intervention but did lead to the development of an innovative approach to measure service integration. The Integration or “Integra” Index was developed to address the challenges, by providing an independent measure of integration at each facility over time (Mayhew et al. 2016). Where clients repeatedly visited clinics scoring highly for “functional” integration (i.e., all clients were able to receive, and some actually did receive, integrated care each day the clinic was open over a week), statistically significant improvements were found in HIV testing rates, reported consistent condom use, and technical quality of care of integrated FP‐HIV and HIV‐postnatal services (Warren, Abuya, and Askew 2013; Integra Initiative 2015c; Church et al. 2017; Mutemwa et al., forthcoming).

The Integra evaluation also demonstrated that although having integrated physical structures, equipment, and supplies, and trained staff are all important, just having these components does not automatically ensure that clients will receive integrated services (Mayhew et al. 2016). Motivated staff must initiate more than one service or a client must demand more than one service during a consultation. Evidence from Integra suggests that aside from formal structural changes, building a health‐sector workforce with the agency and authority to work flexibly to make decisions, communicate effectively throughout services (and across sectors), and share workloads as part of a team is critical to sustainable programs of integrated SRH‐HIV care (Mutemwa et al. 2013; Ndwiga et al 2014; Integra Initiative 2015a; Colombini 2016a; Mayhew et al. 2017).

Finally, Integra sought to explore HIV‐positive clients’ experience of stigma in a variety of settings, both service‐based (when seeking FP, antenatal, and postnatal care services) and facility‐based (when seeking services in different facility types). Findings indicate a complex relationship between stigma and service integration (Church et al. 2013; Colombini et al. 2014). Qualitative data from Integra suggest that clients at sites providing HIV‐only care felt greater confidentiality, knowing that those around them were HIV‐positive, with support gained from other clients (Church et al. 2013; Colombini et al. 2014), while others felt that “blending in” at a more integrated site was preferable (Mulrenan et al. 2015). In other circumstances, clients attending HIV services at integrated or partially integrated clinics have perceived increases in stigma or risk of stigma due to factors such as nonconfidential staff practices (Church et al. 2014; Colombini et al. 2014).

The second major piece of research on SRH‐HIV service integration was the FACES project. This was conducted by the University of California, San Francisco (UCSF) and the Kenya Medical Research Institute (KEMRI) and primarily focused on integrating FP into HIV care and treatment in Western Kenya, including whether FP‐HIV integration improved uptake of more effective FP methods (oral contraceptives, implants, IUDs, and sterilization) as well as reducing unintended pregnancies (Grossman et al. 2013). The FACES project demonstrated that integration of FP services into HIV care centers increased use of more effective contraceptive methods with a (nonsignificant) reduction in condom use (Grossman et al. 2013). The project also demonstrated that integration of FP and HIV services led to increased use of more effective contraception (i.e., hormonal and permanent methods, and intrauterine devices) and decreased pregnancy rates (Cohen et al 2017).

More recently, the project's focus has been on the effects of integration on gender dynamics and men's and women's perceptions and decisions about using FP, as well as provider perceptions of integrated FP‐HIV services (Newmann et al. 2015; Onono et al. 2015; Tao et al. 2015; Newmann et al. 2016). Interventions aimed at increasing male partners’ HIV testing have had a positive effect on the uptake of provider‐initiated testing and counseling and should be encouraged (Patel et al. 2014).

In the third major initiative on integration of SRH and HIV, FHI360 led a range of studies on the integration of SRH and HIV services. They demonstrated tangible gains in access to contraception for women living with HIV (including long‐acting and reversible methods of contraception) (Halperin, Stover, and Reynolds 2009; FHI360 2010; Credé et al. 2012; Wilcher et al. 2013a and 2013b). Research on integrating HIV testing and counseling and family planning by community health workers indicated it is feasible and acceptable and provides an opportunity to increase HIV testing among family planning clients (Brunie et al 2016). In a study evaluating male engagement in integrated FP and HIV service use, health‐seeking practices improved for men, especially condom use, HIV testing, and health facilities visits (Ghanotakis et al. 2016). Integrated services also played a role in the prevention of mother‐to‐child transmission of HIV—specifically by promoting access to FP and by understanding key populations’ SRH needs (FHI360 2013; Ippoliti 2017 in this issue).

## ARTICLES IN THIS SPECIAL ISSUE

This special issue builds on this growing body of research on SRH‐HIV integration, and extends the discussion to wider issues within the SRH and rights agenda. Our purpose is to bring together critical reviews and original research within a broader understanding of integration (i.e., not disease‐specific but more person‐centric) and discuss the gaps emerging from the research that need to be addressed. The articles in the issue address: 1) more recent evidence on models of integrating FP within HIV treatment services, 2) the impact of exposure to integrated services on HIV testing in Kenya, 3) integrated response to intimate partner violence, 4) key populations’ reproductive health needs, and 5) pregnancy experiences of female sex workers in Ethiopia.

### Models of Integrating Family Planning Services into HIV Care

In this issue, Harberlen and colleagues describe the challenges women living with HIV encounter in accessing FP services. In 2014, the Salamander Trust found that 60 percent of women living with HIV had at least one unintended pregnancy, and less than half had ever used contraception. Unmet need for contraception is high in this population, with 66–92 percent of women reporting not wanting another child (now or ever), but only 20–43 percent using contraception (Credé et al. 2012; Sarnquist, Rahangdale, and Maldonado 2013). HIV‐positive women are also more likely to have an unintended pregnancy than HIV‐negative women (Schwartz et al. 2012; Baumgartner 2014; Kimani et al. 2015a). Few studies have focused specifically on the fertility desires, contraceptive needs, and FP behaviors of HIV‐positive women after childbirth, yet the risks of women living with HIV becoming pregnant again are higher than among women who are HIV‐negative (Rutenberg and Baek 2005; Warren, Abuya, and Askew 2013; Kimani et al. 2015b). Programmatic focus on condoms within HIV treatment settings is problematic (Warren, Abuya, and Askew 2013; Church et al. 2014; Kimani et al. 2015a). While it is encouraging that many clients are motivated to use condoms, capacity to sustain their use as a long‐term contraceptive option is often more limited (Beyeza‐Kashesya et al. 2011). In this issue, Haberlen and colleagues review the evidence on positive and negative outcomes of integrated FP‐HIV care and treatment models between 1990 and 2016, including contraceptive prevalence, unmet need for FP, unintended pregnancies, client knowledge of and attitudes toward FP among women living with HIV, client satisfaction and service quality, and costs and cost‐effectiveness. They find limited evidence of change in unmet need, and although access to FP improved the method mix—specifically access to noncondom modern methods—this was not always sufficient (Kosgei et al. 2011). It is clear that HIV service providers need to receive regular updates and capacity building to be able to advise clients on the delicate and often inseparable balance of infection and pregnancy risks faced by clients (e.g., through provision of educational materials), with back‐up methods of contraception including dual‐method use (condom and modern method) made available. If they are to meet their fertility goals, women living with HIV who seek to delay childbirth or limit their family size should be offered a broader method mix, including long‐acting reversible contraceptives (LARCs) that they find acceptable (Kimani et al. 2015a; Kakaire et al. 2016).

### Impact of Integrated Services on HIV Testing

In medium‐ and high‐HIV environments, WHO (2007) recommends repeated HIV testing among at‐risk sexually active individuals every six months to one year. In this issue, Church and colleagues assess the impact of the integrated HIV and FP service model on the HIV testing and counseling (HTC) uptake among FP clients in Kenya, where HIV prevalence is around 6 percent. Researchers tested the effect of four different exposure definitions of integrated care: an intervention involving training and reorganization; receipt of both RH and HIV services at recruitment; a functional measure of facility integration at recruitment (using the Integra Index measures); and a woman's cumulative exposure to functionally integrated care across different facilities over time (again using the Integra Index). Church and colleagues discovered that high cumulative exposure to integrated care over two years had a significant effect on HTC goals among a cohort of FP clients. The integrated delivery approach of postnatal services was also beneficial in increasing provider‐initiated testing and counseling for HIV and long‐acting FP services among postpartum women (Kimani et al. 2015b). Other Integra work underscores that context is a critical determinant of integration practices for provision of HTC within an FP clinic, and programs must move beyond simplistic training and equipment provision if integrated care interventions are to be sustained (Church et al. 2015).

### Integrated Response to Intimate Partner Violence

In this issue, Columbini, Dockerty, and Mayhew make a valuable contribution to the literature, since violence is a risk factor for HIV, and comprehensive SRH‐HIV responses would necessarily consider how to respond to intimate partner violence. The authors’ focus on the systems dimensions of service integration is an important addition, as it is a neglected area of violence‐response research (Colombini, Mayhew, and Watts 2008; Colombini et al. 2012; Garcia‐Moreno et al. 2015). Given the paucity of evaluated integration approaches to intimate partner violence, the article offers a synthesis of studies on health‐sector responses to intimate partner violence in low‐ and middle‐income countries, and discusses the barriers and facilitators of integrated and less integrated services.

Colombini and colleagues find that some facilitators for integrating discussions on intimate partner violence are especially important. These include management support, clear policies and protocols, and trained providers with empathetic attitudes. Qualitative Integra work also reveals the role health services could make in supporting violence prevention within integrated SRH‐HIV services (Colombini et al. 2016b), in particular the importance of integrating discussions on risks for partner violence and fear of disclosure into HIV counseling and testing, helping women develop communication skills for disclosing their status, and reducing fear about marital separation. It highlights the need for providers to consciously help reduce the blame for HIV transmission and raise awareness of HIV as a chronic disease.

### Meeting the SRH Needs of Key Populations

In this issue, Ippoliti, Nanda, and Wilcher examine FP, reproductive health services, and key populations—including young sex workers—and the underlying determinants of these populations’ vulnerability to poor reproductive health outcomes and their obstacles to accessing reproductive health services. It is estimated that 40–50 percent of all new HIV infections among adults worldwide occur among key populations, such as female sex workers, men who have sex with men, people who inject drugs, transgender people, and prisoners (UNAIDS 2014), but relatively little attention has been paid to these key populations’ reproductive health needs. Key populations rarely access mainstream SRH services but do access HIV and specialized services, and an integrated service approach based upon their needs would benefit these disadvantaged groups. Too often the focus is on HIV prevention and not on how SRH issues—including pregnancy, both intended and unintended—affect their lives. This article describes what is currently known about the reproductive health of female key populations affected by HIV, including their pregnancy intentions, FP use, and abortion practices. Secondly, it identifies the barriers preventing female key populations from realizing their reproductive intentions. And thirdly, it recommends approaches for improving female key populations’ reproductive health. Few studies look at the reproductive health needs of partners of this group, and no studies were found on the reproductive health or FP needs of transgender people.

### Pregnancy Experiences of Female Sex Workers

The high prevalence of unintended pregnancies and abortion among sex workers is well documented in the literature. While the integration of FP and HIV programming makes sense for sex workers, in this issue Yam and colleagues examine the circumstances of pregnancy and childbirth among women selling sex, to understand the complex SRH needs of this very vulnerable group. This study, in Ethiopia under the Link Up project, provides an in‐depth understanding of the issues sex workers face and examines pregnancy intentions, circumstances surrounding unintended pregnancies, contraception use at time of pregnancy, fathers, selling sex while pregnant, abortion experiences, pregnancy among female sex workers living with HIV, and the commonalities and differences of pregnancies before and after sex work.

## FUTURE DIRECTIONS

While there is no blueprint for integration, more can be done in utilizing SRH as the entry point for reducing unmet need for HIV testing, treatment, care, and support services by increasing the range of services available and accessed in one visit to a health facility. This special issue demonstrates that much has been achieved since the Glion Call to action in 2004, but challenges continue with integrating HIV and SRH services—two obviously interrelated programs. Evidence suggests the potential of improved productivity through integration, but at the same time there are significant challenges with the pace of productivity gain (Sweeney et al. 2014). Progress toward providing universal health care requires changing how health systems interact with communities and clients, and incorporating integration as a core principle in service delivery. This includes infrastructure, equipment, and commodities, and a skilled, efficient health workforce (in sufficient numbers) at all levels, but primarily at the community and first level of care. Moreover, efforts to implement integration need to be assessed within the broader context of human resource planning to ensure that neither staff nor clients are negatively affected by integration policy, and that providers’ concerns about staffing, training, and commodity stock‐outs are addressed (Mutemwa et al. 2013; Wilcher et al. 2013a; Ndwiga et al. 2014; Sweeney et al. 2014; Newmann et al. 2015). These health‐systems obstacles need to be resolved to enable scale‐up and sustainability of cost‐effective, integrated service provision (Wilcher et al. 2013a; Newmann et al. 2015; Mayhew et al. 2016). Stewardship and management systems by mid‐level managers for both resources and supporting health providers’ agency, and flexible decision‐making for client‐centered, integrated care, especially for women living with HIV, are essential (Colombini et al. 2016a).

One area of increased focus in recent years is men's perceptions of integrated HIV and SRH use, and how gender plays a role in care‐seeking behavior. Interventions aimed at increasing male partners’ HIV testing have demonstrated a positive effect on the uptake of provider‐initiated testing and counseling among women (Kimani et al. 2015b). Integration of FP‐HIV services can have a broad impact on the majority of women and men accessing HIV care and treatment who may be interested in practicing contraception (Newmann et al. 2013). Men continue, however, to avoid HIV and SRH services because of the stigma associated with attending health services with mainly female providers, as well as their preference for traditional healers who are mainly male (Mak 2016). More research is required to understand how improved gender equity might be leveraged to increase access to HIV testing and treatment, contraceptive use, and other reproductive health outcomes (Hawkes and Buse 2013; Onono et al. 2015; Ghanotakis et al. 2016; Newmann et al. 2016; Pascoe, Peacock, and Dovel 2016).

Global policies have broadened the scope of integration to include gender‐based violence (GBV) prevention and promotion of a stigma‐free environment (IATT 2011). The inclusion of prevention of GBV and stigma as essential components of SRH services demonstrates that a safe and supportive environment is essential in health‐service delivery to improve health outcomes (Haberlen and colleagues, in this issue), though interventions are still required to mitigate felt stigma (Russell et al. 2016). While broader changes are needed to resolve gender disparities, practical steps can be institutionalized within health facilities to reduce, or avoid increasing, intimate partner violence among women with HIV (Mulrenan et al. 2015). Precisely strategized confidentiality policies and skilled management of the integration process are critical for a successful integration program that does not increase either perceived or enacted stigma (Church et al. 2014; Integra Initiative 2015b). Ippoliti, Nanda, and Wilcher (in this issue), and Yam et al. (in this issue) describe the gaps that remain in addressing key populations’ SRH needs—beyond HIV risks and behaviors—and how to understand the complexity of context. Facilitators that enhance a comprehensive response to intimate partner violence include critical health system inputs, such as management support, clear referral options, skilled, empathetic providers, and clear guidelines.

Broadening the constellation of services for women and men is heartening to see. However, each individual must be offered integrated or comprehensive services based on their health needs and no one should experience stigma or discrimination when accessing these services. Women often discover their HIV status when they are pregnant, but a gap remains in the continuum of care, with women living with HIV not receiving a comprehensive postnatal package of care addressing their SRH needs, meeting their fertility desires, or ensuring healthy motherhood (Warren, Abuya, and Askew 2013). Integrated services offering early detection and treatment for cervical cancer screening is both feasible and acceptable, and should be regarded as an essential part of comprehensive SRH and HIV care (Odafe et al. 2013; Huchko et al. 2015).

There are still gaps in both provision of care and research, and there continues to be limited availability of long‐acting contraception for those who need it. Unmet need for postpartum FP remains high (61 percent) (Moore et al. 2015). Pregnancy risks can peak at 6 to 11 months after childbirth, and women often rely on short‐term methods only (Moore et al. 2015). Men and women living with HIV continue to be offered short‐term methods, with an overreliance on condoms (Church et al. 2014), and counseling on condom use is often the only contraceptive information provided. It has been demonstrated, however, that women living with HIV find long‐acting methods acceptable, with high satisfaction rates (84–90 percent) (Dhont et al. 2009; Kakaire et al. 2016).

Initiatives such as the SRHR and HIV Linkages project in seven countries in Southern Africa (UNFPA and UNAIDS 2015) show increased impetus for scaling up SRH and rights and HIV integration, especially linking SRH and rights and HIV at the policy, health system, and service‐delivery levels. Governments elsewhere are promoting integration but have not ensured SRH and HIV strategies and policies are aligned accordingly, leading to an ad hoc approach to integrated service delivery and placing a greater burden on frontline health workers (Hope et al. 2014; Hopkins and Collins 2017). Further research on integrated SRH‐HIV services within health systems is urgently needed to inform the planning, financing, and coordination of integrated service delivery (Hope et al. 2014). Where integration policies exist and stakeholders develop plans to scale up and strengthen SRH‐HIV service integration, it is critical to include an implementation research component to evaluate how best to scale up integrated service delivery. This will overcome the need for countries to scale up using a trial‐and‐error approach. Integra was the first large‐scale implementation science research on HIV integrated into SRH service delivery, and many lessons were learned, both about what do to and what to avoid, including the integration index measuring the extent to which any given facility is integrated (Mayhew et al. 2016).

Integra and other studies have grappled with the link (or lack thereof) between integration of SRH‐HIV service and unintended pregnancies. There is limited evidence—with challenges in different methodologies—on whether an integrated counseling session can change women's (mainly) sexual and reproductive behavior. This research gap needs to be filled.

## CONCLUSION

SRH services are regarded as an example of a broader platform for health‐service delivery that is not disease‐specific, but provides a person‐centric, holistic approach to health and well‐being that could contribute to the wider discourse on the opportunities and known challenges for integrating health services. The evidence base, not only on why to integrate SRH and HIV services but also on how to integrate them, has expanded considerably over the past decade, with several large‐scale research initiatives. Nevertheless, gaps remain, especially on how to effectively and cost‐efficiently scale up integrated service delivery, whether and how to incorporate other related services like violence response, and how best to reach vulnerable key populations. The articles in this special issue provide valuable contributions to update the evidence base for SRH‐HIV integration and delineate key areas for future research in this field.
